# Material and Microstructure Analysis of Wood Color Paintings from Shaanxi Cangjie Temple, China

**DOI:** 10.3390/molecules29122734

**Published:** 2024-06-08

**Authors:** Dan Huang, Kezhu Han, Gele Teri, Cong Cheng, Yunpeng Qi, Yuhu Li

**Affiliations:** Engineering Research Center of Historical Cultural Heritage Conservation, Ministry of Education, School of Materials Science and Engineering, Shaanxi Normal University, Xi’an 710119, China; hdansnnu@163.com (D.H.); hankekezhu@126.com (K.H.); terigelesnnu@163.com (G.T.); congcheng2017@snnu.edu.cn (C.C.)

**Keywords:** CangJie temple, color paintings, pigments and binders, Raman spectra, FTIR spectra, Py-GC/MS

## Abstract

Cangjie Temple was built to commemorate Cangjie, the legendary inventor of Chinese characters. It stands as one of the few remaining temples in China dedicated to the invention and creation of writing. In this study, the material properties of wooden paintings from the Cangjie temple were characterized using Polarized Light Microscopy (PLM), Scanning Electron Microscopy coupled with Energy Dispersive X-ray Spectroscopy (SEM-EDS), Micro-confocal Raman Spectroscopy, X-ray Diffraction (XRD), Fourier Transform Infrared Spectroscopy (FTIR), and Pyrolysis-Gas Chromatography-Mass Spectrometry (Py-GC/MS). It was confirmed that the pigments of the paintings included cinnabar, lapis lazuli, lead white, Paris green, and carbon black. The proteinaceous glue was used as an adhesive in the pigment samples, with tung oil likely being utilized as a primer for the wooden structures before painting. This study not only provides valuable data support for the conservation and restoration of the architectural features of Cangjie Temple but also provides useful reference for the maintenance and inheritance of similar ancient buildings.

## 1. Introduction

**Figure 1:**
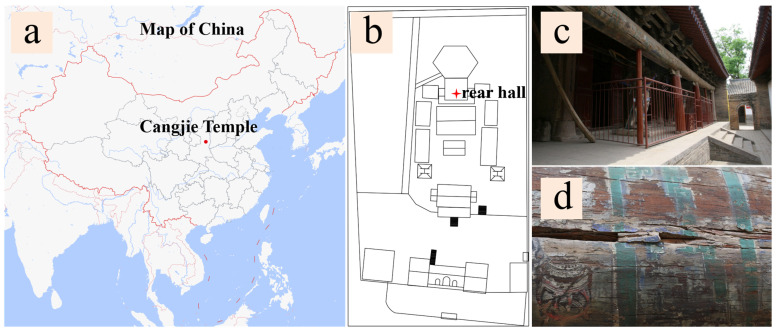
CangJie temple: (**a**) the location of CangJie temple (the red dot); (**b**) the floor plan of CangJie temple; (**c**) the color painting executed on the wooden beams in the rear hall of Cangjie Temple; (**d**) partially painted wooden beams.

The Cangjie Temple, located approximately 35 km northeast of Baishui County, Weinan City, Shaanxi Province (Figure 1a), was established to commemorate Cangjie, the legendary inventor of Chinese characters. The exact date of the temple’s establishment remains unclear; however, according to the “Cangjie Temple Stele”, by the fifth year of the Yanxi era during the Han Dynasty (162 AD), the temple was already well-developed and had a history spanning over two millennia. The rear hall of the temple has a 16-m-long, 55-cm-diameter wormwood log, and the color paintings are drawn on it (Figure 1b–d). These murals, as physical relics of history, possess significant historical and artistic value. They also carry substantial information about traditional culture. The form and content, purpose and function, materials and physicality, location and surroundings, tradition and technique, as well as the spirit and emotions conveyed by these paintings, constitute essential aspects of cultural heritage. These elements reflect the characteristics of mural art in the Central Plains region and the underlying social and cultural customs and traditional concepts. To better preserve these paintings, it is crucial to conduct a comprehensive evaluation of the various components of the paintings.

The mural paintings of ancient architecture are composed of three layers from the interior to the exterior: wooden components, the ground layer, and the painting layer [1]. The painting layer consists mainly of pigments and binder materials [2]. The pigments used are mainly mineral pigments, supplemented by plant pigments, and mixed with binders [3]. The most common mineral pigments include cinnabar (HgS), iron oxide red (Fe_2_O_3_), lead red (Pb_3_O_4_), orpiment (As_2_S_3_), realgar (As_4_S_4_), malachite (Cu_2_(OH)_2_CO_3_), azurite (Cu_3_(CO_3_)_2_(OH)_2_), atacamite (Cu_2_(OH)_3_Cl), calcite (CaCO_3_), lead white (PbCO_3_), carbon black, and ferroferric oxide (Fe_3_O_4_) [4,5]; while the plant pigments include gamboge (yellow), indigo (blue), carmine (red), and cyanine (green), etc. [6,7]. The ground layer is primarily composed of materials such as pig blood, lime, flour, and tung oil, which are mixed [8]. This composition facilitates the adherence of mural pigments and prevents some chemical reactions with the pigment layer [9].

The analysis of pigment plays a crucial role in the conservation of ancient architectural paintings. A variety of analytical techniques have been employed for characterizing the inorganic materials of the paintings, including scanning electron microscopy (SEM) coupled with energy dispersive X-ray analysis (EDS), polarized light microscopy (PLM), X-ray fluorescence spectroscopy (XRF), X-ray diffraction (XRD), Raman spectroscopy, Fourier transform infrared spectroscopy (FT-IR), and gas chromatography or liquid chromatography coupled with mass spectrometry (Py-GC/MS) [10,11,12,13]. For instance, Zelinská, J. et al. [14] used optical microscopy, XRF, SEM-EDS, Raman spectroscopy, and FT-IR to identify chalk, cinnabar, lead-tin yellow, cerussite (lead white), malachite, azurite, an iron oxide, and fluorite. M.L. Franquelo et al. [15] utilized portable XRF, μ-XRD, SEM-EDS, μ-FTIR, and μ-Raman instruments to analyze the multicolored wooden sculpture “Saint Anne with the Virgin and Child”. Cheng Xiaolin et al. [16] applied Raman Spectroscopy in conjunction with XRD to identify green pigment containing copper and arsenic elements from various regions. Deborah Lau et al. [17] characterized paint layers in micro-samples using Environmental SEM-EDS and Raman Spectroscopy imaging.

Adhesives play a crucial role in the stable preservation of painted surfaces, serving not only as the medium binding pigment particles together but also as the carrier that adheres the paint layer to the substrate surface [18]. Consequently, the analysis of adhesives is indispensable. The binding materials can be categorized into proteins (such as egg white, pigskin glue, and fish glue), polysaccharides (such as peach gum and gum arabic), oils (tung oil, linseed oil, and castor oil), and others (such as rosin, beeswax, and bitumen) [19]. These materials may be used alone or in combinations of two or even three types. Common analytical methods for adhesives include nuclear magnetic resonance (NMR) [20], Raman spectroscopy [16], mass spectrometry [21], infrared spectroscopy (IR) [22], micro-fluorimetry [23], and enzyme-linked immunosorbent assay (ELISA) [24]. Among these, pyrolysis-gas chromatography/mass spectrometry (Py-GC/MS) is currently the most frequently employed method for the analysis of binding materials [25].

In this study, a comprehensive analysis was conducted on the pigment layer and earth layer, using PLM, SEM-EDS, and Raman spectroscopy, to identify the chemical composition, determine the mineral composition of wood by XRD, and identify functional groups with FTIR. Also, Py-GC/MS was employed to analyze thermal decomposition products, to determine the use of adhesives in the construction process of Cangjie Temple. These findings not only provide data support and theoretical basis for the current protection and restoration of Cangjie Temple but also offer valuable references for the conservation of similar cultural heritage.

## 2. Results and Discussion

### 2.1. Cross Section

**Figure 2:**
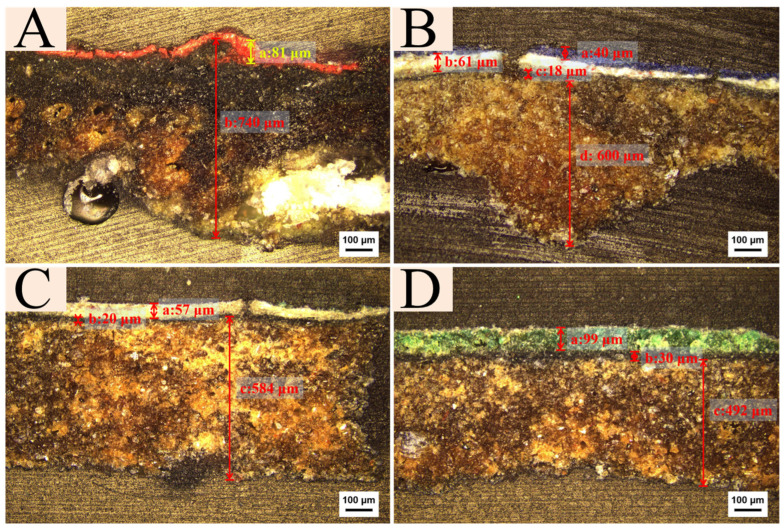
Cross sectional PLM images of samples. (**A**). Sample Y1, (**B**). Sample Y2, (**C**). Sample Y3, and (**D**). Sample Y4.

The cross-sectional of the investigated samples were observed under PLM, as shown in Figure 2. All samples are composed of several layers of different colors and compositions, superimposed on the ground layer. The cross-sectional structure of sample Y1 (Figure 2A), consists of a single pigment layer. The thickness of the red pigment layer is uneven, reaching a maximum of approximately 81 μm, while the ground layer is about 700 μm in thickness. The cross-sectional structure of sample Y2 (Figure 2B), reveals a multi-layered pigment composition. From the innermost to the outermost, the layers comprise a black layer, a white layer, and a blue layer. The thickness of the black pigment layer is approximately 18 μm, the white pigment layer measures about 61 μm, and the blue surface layer is around 40 μm, with the ground layer’s thickness approximating 600 μm. The cross-sectional structure of sample Y3 (Figure 2C), features a two-layered pigment arrangement. The outer white pigment layer is about 57 μm, whereas the inner black pigment layer measures approximately 20 μm, with the ground layer’s thickness at roughly 584 μm. The cross-sectional structure of sample Y4 (Figure 2D), also exhibits two-layered pigments. The thickness of the outer green pigment layer is approximately 99 μm, the thickness of the inner black pigment layer is about 30 μm, and the ground layer (approximately 492 μm in thickness).

The cross sections of sample Y2 include the ground layer, black base layer, white layer, and pigment layer, whereas sample Y1 applies red directly onto the ground layer without a black base layer. As for samples Y3 and Y4, the cross sections include the ground layer, black base layer, and pigment layer. Overall, the composition and technique of the various patterns found in the colored paintings of Cangjie Temple can be described as involving wooden support, the application of ground material to make the wood surface flat, the use of black pigment to enhance the base for painting, and the final stages of painting. The material thickness of each layer should be tailored to the specific characteristics of the wall and the desired aesthetics.

### 2.2. Analysis of the Pigments

#### 2.2.1. Red Pigment

**Figure 3:**
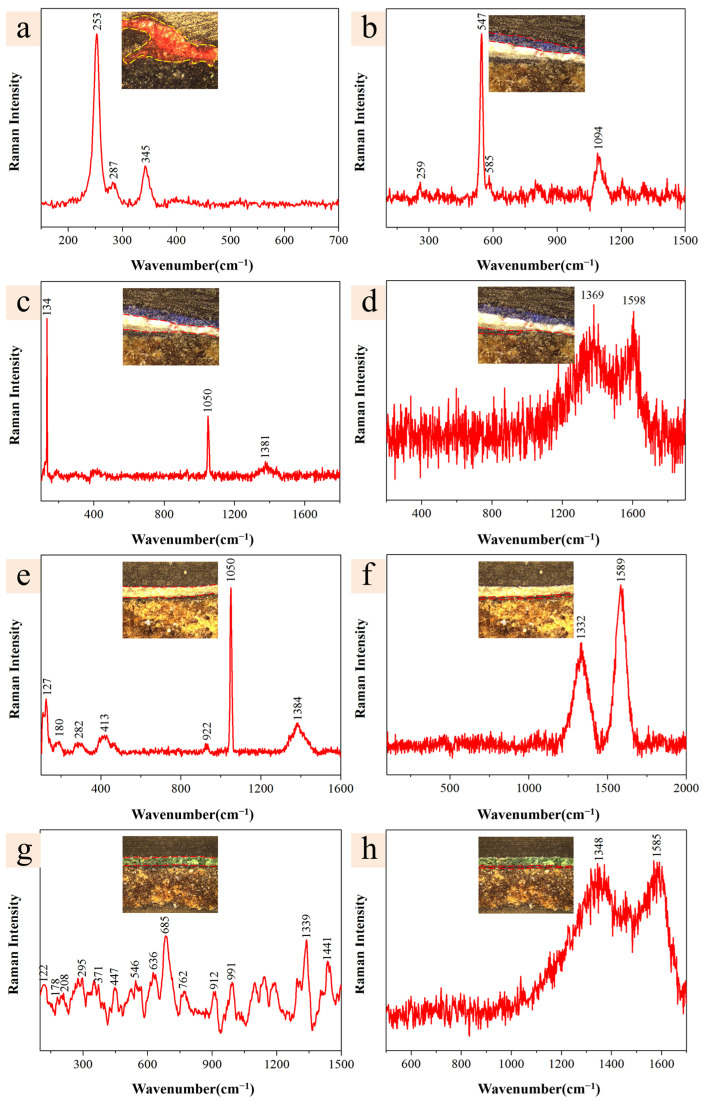
Raman spectra of the different layer samples (yellow and red frames - Raman spectra collection area of sample): (**a**) red of Y1; (**b**) blue of Y2; (**c**) white of Y2; (**d**) inner black of Y2; (**e**) white of Y3; (**f**) inner black of Y3; (**g**) green of Y4; and (**h**) inner black of Y4.

EDS analysis, as presented in Table 1, revealed the elemental composition of the red pigment to include C, O, Al, Si, Hg, S, K, and Ca, without detection of Pb and Fe. This composition led to the assumption that the red pigment is cinnabar (HgS) [26]. To further confirm the composition of the red pigment, laser Raman spectroscopy was employed for characterization. As presented in Figure 3a, the predominant Raman peaks were observed at 253 cm^−1^, 287 cm^−1^, and 345 cm^−1^. The most intense peak at 253 cm^−1^ is attributed to the α1 stretching vibration of characteristic Hg–S, and the peaks at 287 cm^−1^ and 345 cm^−1^ correspond to the stretching vibrations of Hg-S [27], confirming the pigment as cinnabar. This finding is consistent with the results obtained from spectral analysis. 

Cinnabar, as a red mineral pigment, occupies a significant position in ancient Chinese architectural painting. In addition to serving as an element for enhancing color, it is valued for its unique antibacterial and insect-repellent properties, effectively preventing the decay and insect damage of wood, thereby prolonging the preservation lifespan of ancient buildings [28]. The application history of cinnabar dates back to ancient times, with its earliest traces found in colored pottery unearthed from the Dadiwan site in Qinan, Gansu Province, dating back approximately 7000 years ago to the Neolithic era [29]. As history evolved, cinnabar found widespread application in architecture, painting, and religious rituals in different periods. Especially during the Qing Dynasty, cinnabar was extensively used in the painted decorations of buildings such as the Puren Temple [30] and the Beiqing Mosque [31], showcasing its unique artistic charm and practical value.

#### 2.2.2. Blue Pigment

EDS analysis, as presented in Table 1, indicated that the primary elements of the blue pigment include C, O, Na, Al, Si, S, and Ca, with Cu, Fe, and Ba not detected, leading to the hypothesis that the blue pigment was lapis lazuli, also known as ultramarine [(Na, Ca)_8_(AlSiO_4_)_6_(S, Cl)_2_] [32,33]. The laser Raman spectroscopy of the blue pigment’s surface, shown in Figure 3b, exhibited characteristic peaks at 259 cm^−1^, 547 cm^−1^, 585 cm^−1^, and 1094 cm^−1^, in line with the characteristic Raman peaks of lapis lazuli. The peaks at 547 cm^−1^ and 585 cm^−1^ were attributed to the symmetric stretching vibrations of S^3−^ and S^2−^ ions [34], respectively; the absorption peak at 1094 cm^−1^ was associated with the stretching vibration of Si–O–Si [35]. Lapis lazuli has been used as a blue pigment for painting in ancient China, as evidenced in the murals of the Mogao Caves in Dunhuang, the Yulin Grottoes in Anxi, the Maiji Mountain Grottoes in Tianshui, and the Bingling Temple Grottoes in Yongjing [36]. Ultramarine pigment is an inorganic pigment, that was artificially synthesized in the Western world in the 1830s, and during the late Qing Dynasty, synthetic ultramarine was introduced to China [31]. Due to its affordability and accessibility on the market, it was widely used in the fields of painting and restoration of colored artworks. Examples of its usage include the Beiqing Mosque [31], the Summer Palace, and the Dagaoxuan Temple of the Imperial Palace in Beijing [28].

The primary elements of the white pigment layer beneath the blue pigment were identified as C, O, and Pb, with Ca, S, and Cl not detected, suggesting that the white pigment might be lead white [37]. The laser Raman spectra, as shown in Figure 3c, present characteristic peaks at 134 cm^−1^, 1050 cm^−1^, and 1381 cm^−1^, corresponding to the standard Raman spectrum of lead white. The peak at 1049 cm^−1^ was attributed to the symmetric stretching vibration of the carbonate ion (CO_3_^2−^) [38,39]. Lead white is one of the earliest known white pigments produced through artificial methods, and it has been widely used since around 400 BC [31]. In the architecture of the Jiangxue Palace within the Forbidden City in Beijing, China [40], lead white has been utilized as a crucial white pigment. Similarly, there are records of its usage in certain parts of the Altar of Agriculture of Beijing, China [28].

The main elements of the black pigment at the base layer were C, O, Al, Si, S, Pb, and Ca. Common black pigments used in ancient architectural paintings include carbon black, iron black, lead dioxide, and black cinnabar, with carbon black being particularly widespread, derived from graphite minerals or various inks produced from the ash of burnt plant or animal fats [28,41]. The laser Raman spectra of the base black pigment, illustrated in Figure 3d, presented two broad peaks at 1369 cm^−1^ and 1598 cm^−1^, consistent with the Raman signature peaks of carbon black [42,43]. The peaks at 1348 cm^−1^ and 1585 cm^−1^ were assigned to the D and G bands of v(C–C) and v(C=C) vibrations [44,45,46,47], respectively, confirming the black pigment as carbon black. 

Carbon black, primarily composed of amorphous carbon, boasts a relatively simple manufacturing process, contributing to its widespread application in fields such as painting, documentation, and calligraphy [31]. In ancient China, carbon black was used as a common back pigment in numerous architectural paintings due to its color stability and strong durability, effectively showcasing the style and characteristics of ancient buildings. For instance, carbon black was utilized in ancient buildings such as the Xianqing Temple in Shanxi [35] and the Royal Palace of the Taiping Heavenly Kingdom [45].

#### 2.2.3. White Pigment

EDS analysis, as presented in Table 1, indicated that the white pigment comprises elements such as C, O, Mg, Al, Si, and Pb, with the absence of Ca, S, and Cl, suggesting that the white pigment might be lead white [37]. The laser Raman spectrum of the pigment, depicted in Figure 3e, showed characteristic peaks at 127 cm^−1^, 180 cm^−1^, 282 cm^−1^, 413 cm^−1^, 922 cm^−1^, 1049 cm^−1^, and 1384 cm^−1^. Notably, the peak at 1049 cm^−1^ was attributed to the symmetric stretching vibration of the carbonate ion (CO_3_^2−^) [38], and the peak near 413 cm^−1^ was due to the vibration of the Pb–O bond [48]. In ancient China, carbonate minerals used as white pigment or coatings typically exhibit a strong Raman peak around 1050 cm^−1^, usually indicative of lead white (2PbCO_3_·Pb(OH)_2_) or cerussite (PbCO_3_), with lead white being more common [49]. Studies have shown that the Raman spectra of lead white and cerussite differ significantly in the low wavenumber range (100~500 cm^−1^); lead white exhibits a distinct Raman peak at 415 cm^−1^, which was absent in cerussite [38,50], thus confirming the pigment as lead white.

The primary elements of the black pigment in the base layer include C, O, Mg, Al, Si, S, and Pb, as shown in Table 1. The black pigment is hypothesized to be either lead dioxide or carbon black. The laser Raman spectrum of the black pigment layer, presented in Figure 3f, with characteristic peaks at 1332 cm^−1^ and 1589 cm^−1^, aligns with the Raman signature peaks of carbon black [44,45], thereby confirming the black pigment as carbon black.

#### 2.2.4. Green Pigment

EDS analysis, as presented in Table 1, revealed the principal elements of the green pigment to be C, O, Cu, and As, suggesting the pigment could be Paris green (Cu(CH_3_COO)_2_·3Cu(AsO_2_)_2_), cornwallite (Cu_5_(AsO_4_)_2_(OH)_4_), or malachite (CuCO_3_·Cu(OH)_2_) [51]. The laser Raman spectrum of the pigment, shown in Figure 3g, exhibits characteristic peaks at 122, 178, 208, 295, 371, 447, 546, 636, 685, 762, 912, 991, 1339, and 1441 cm^−1^, corresponding with the Raman spectrum of Paris green [52]. Notably, the multitude of bands within the 400 to 100 cm^−1^ range can be attributed to the vibrations of Cu–O and As–O; the peak at 1441 cm^−1^ corresponds to the acetate groups (–COOH) present in Paris green [40]. Thus, the surface green pigment is identified as Paris green. 

Paris green, synthesized from copper (II) acetate and arsenic trioxide, has been widely used in architectural mural painting in China since its introduction from Europe, owing to its unique color and stability. For example, the eave murals of the Puren Temple of the Qing dynasty (in 1713 AD) [30], and the Dagaoxuan Temple of the Imperial Palace, Beijing [28], both employed Paris green in their compositions.

The primary elements of the black base pigment are C and O, as indicated in Table 1, leading to the hypothesis that the black pigment might be carbon black. The laser Raman spectrum of the black pigment layer, shown in Figure 3h, features two broad peaks at 1348 cm^−1^ and 1585 cm^−1^, consistent with the Raman signature peaks of carbon black [44,45], thereby confirming the black pigment as carbon black.

### 2.3. Analysis of Inorganic Fillers and Adhesives

**Figure 4:**
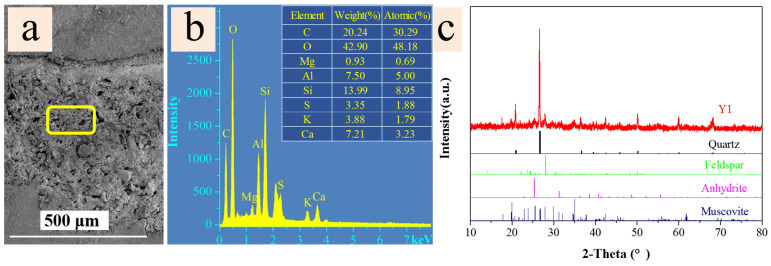
(**a**) SEM image (yellow square—EDS spectra collection area), (**b**) EDS spectra, and (**c**) XRD pattern of the ground layer of sample Y1.

The ground layer of ancient architectural paintings typically comprises a mixture of inorganic substances such as brick dust and lime, combined with organic materials including tung oil, animal blood, and flour, both of which contain proteins [40]. SEM-EDS analysis stands as a notably sensitive technique adept at detecting trace elements, even when present in minute quantities. Therefore, it was chosen for the assessment of inorganic constituents within the ground layer. As shown in Figure 4a,b, the SEM image and EDS spectra of sample Y1’s ground layer, reveal the presence of various elements including C, O, Mg, Al, Si, S, K, and Ca within the ground layer composition. XRD pattern of the ground layer from sample Y1 (Figure 4c) predominantly indicated phases of quartz, feldspar, anhydrite, and muscovite [53,54]. The pigment layer in ancient architectural paintings was constituted of pigment bound by a binding medium, which serves as a fixative that plays a crucial role in the long-term preservation and color stability of the painting [31]. Thus, the analysis of the binder composition is of significant importance.

**Figure 5:**
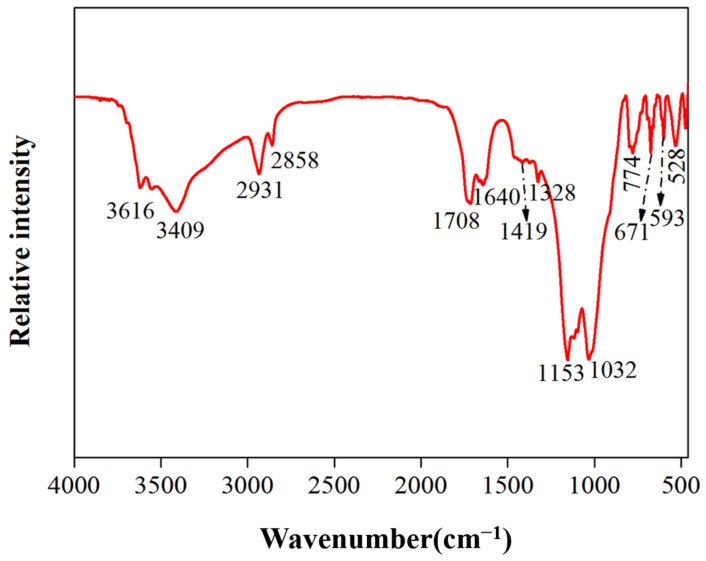
FT-IR spectra of sample Y1.

The FT-IR spectrum of the ground layer, as depicted in Figure 5, displayed characteristic signals of drying oils in the regions of 2950–2850 cm^−1^ (functional group region), 1710–1600 cm^−1^ (double bond stretching region), and 1420–1320 cm^−1^ (double bond deformation region) due to C-H bending [22,55]. Additionally, the presence of N-H stretching vibrations at 3500–3400 cm^−1^ and amide I band (–CONH_2_) at 1640 cm^−1^ (related to C=O stretching vibrations) suggests the presence of proteins in the sample [25,56], indicating that the adhesive components primarily consist of drying oils and proteins.

**Figure 6:**
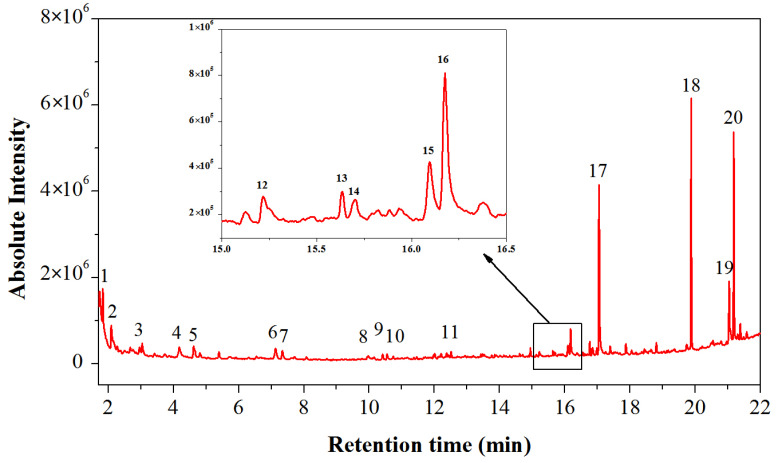
Total ion chromatogram of sample Y1. Peak identification: (1) Acethydrazide, (2) 2-methyl-1-pentanol, (3) Valeraldehyde, (4) Toluene, (5) 1-Octene, (6) Cyclohexanone, (7) Glyceryl methyl ether, (8) 2-Octanone, (9) Cycloheptanone, (10) 6-Heptenoic acid methyl ester, (11) Caprylic acid methyl ester, (12) Glycinamide monohydrochloride, (13) 1-Tetradecene, (14) n-Nonadecane, (15) Methyl 9-Oxononanoate, (16) Dimethyl suberate, (17) Dimethyl azelate, (18) Methyl palmitate, (19) Methyl oleate, and (20) Methyl stearate.

Py-GC/MS analysis of the Y1 sample yielded a total ion chromatogram as shown in Figure 6, and the identified compounds with their retention times and peak areas were summarized in Table 2. The pyrolysis products contained numerous characteristic degradation products of proteins, such as 1-methyl-, Toluene, glycine (found in glue and egg white), and methyl ester [57], indicating that the glue used in these samples contains proteins.

The Py-GC/MS analysis of the sample revealed the detection of dimethyl suberate (peak no. 17), palmitic acid (peak no. 18), oleic acid (peak no. 19), and stearic acid (peak no. 20). Palmitic acid (peak no. 18), oleic acid (peak no. 19), and stearic acid (peak no. 20) were original constituents of drying oils [58], whereas the characteristic peak of dimethyl suberate (peak no. 17) was a distinct marker of tung oil in Py-GC/MS analysis, indicating a product of drying oil polymerization [59]. Linseed oil, walnut oil, poppyseed oil, and tung oil are the most extensively utilized binders in various ancient paintings. Over centuries or even millennia, the unsaturated acids in these drying oils may undergo partial or complete oxidation and polymerization, while the saturated fatty acids remain unaltered, particularly maintaining the ratio of palmitic to stearic acids (P/S) [21,60]. Therefore, the ratio of palmitic acid to stearic acid (P/S ratio) was commonly employed to differentiate various types of drying oils. Specifically, the P/S ratio for boiled tung oil ranges from 0.9 to 1.1, raw tung oil from 1.3 to 1.6, linseed oil from 1.2 to 1.5, poppyseed oil from 1.6 to 1.8, and walnut oil from 1.8 to 2.0 [28]. According to Table 2, the P/S value of palmitic to stearic acid in our Y1 sample was 1.16, further confirming the presence of tung oil components in the sample [61].

## 3. Conclusions

Comprehensive characterization of the pigments and adhesives used in the decoration paintings on the rear hall crossbeams of the Cangjie Temple was conducted utilizing a series of techniques including polarized light microscopy, scanning electron microscopy with energy-dispersive X-ray spectroscopy, laser Raman spectroscopy, X-ray diffraction, infrared spectroscopy, and pyrolysis-gas chromatography-mass spectrometry. The findings reveal that the red pigment employed in the paintings of the rear hall crossbeams is cinnabar, the white pigment is lead white, the green pigment is Paris green, the blue pigment is lapis lazuli, and the black pigment is carbon black, with all analyzed pigments being inorganic mineral pigments.

Cross-sectional images indicate that the red layer comprises a ground layer and a pigment layer; the green and white painting layers consist of a ground layer, an underlayer (black: carbon black), and a pigment layer; the blue pigment, in addition to the blue pigment layer and ground layer, includes two base colors, where the black and white pigments are carbon black and lead white, respectively.

Py-GC/MS and FT-IR analyses reveal the presence of proteins within the ground layer of the pigment samples, as well as the detection of tung oil.

In summary, the analysis of the pigment layers and adhesives in the wooden paintings of the Cangjie Temple provides data support for future conservation and restoration.

## 4. Materials and Methods

### 4.1. Samples

Samples preparation: Samples were collected from the paintings that had fallen off from the rear hall of the Cangjie Temple, which were carefully collected, and sorted using tweezers, to ensure the acquisition of four distinct pigment samples. The selection was confined to diminutive fragments exhibiting hues of red, white, green, and blue. The surface and chromatic states of these samples are delineated in Table 3.

Preparation of cross-section for the pigments: The mold was partially filled with resin (Transparent Cold Mount, Shandong Laizhou, China) at first and the matrix was allowed to solidify for one hour. After this, the samples encompassing both the ground support and the pigment layer were introduced as horizontally as possible onto the resin in order to facilitate its perpendicular cut, and finally, the resin was again filled up to cover all the samples. The preparation was then allowed to solidify for 24 h at room conditions and was cut by a low-speed precision cutting apparatus (DTQ-5, Veiyee, Shanghai, China). The resultant sections were subjected to a sequential polishing regimen using sandpaper grades ranging from 600 to 6000 grit, achieving a uniformly smooth surface.

### 4.2. Experimental Methods and Instrumentations

Polarized light microscopy: The examination of pigment particles and cross-sectional structures was conducted utilizing a polarized light microscope (BX53M, Olympus, Tokyo, Japan), under magnification objectives ranging from 5× to 50×.

Micro-Raman spectroscopy: Pigment analysis was performed with a micro-confocal laser Raman spectrometer (inVia-Reflex, Renishaw, Gloucestershire, UK), employing excitation wavelengths of 532 nm and 785 nm, across a scanning range of 500–4000 cm^−1^. The apparatus was integrated with an optical microscope and analyses were executed utilizing objective lenses of 50× and 100× magnification.

Scanning electron microscopy with energy-dispersive spectroscopy (SEM-EDS): The microstructural observations and elemental content analysis of the samples were carried out using a scanning electron microscope equipped with an energy-dispersive spectroscope (3500U, Hitachi, Tokyo, Japan), operating at an acceleration voltage of 10 kV. The sample surfaces were sprayed with gold, covering a testing range of elements from 11Na to 92U.

X-ray diffractometry (XRD): A high-resolution X-ray diffractometer (Smart Lab, Rigaku Corporation, Tokyo, Japan) was used, utilizing Cu Kα radiation (λ = 1.5406 Å) over a 2θ range of 20–80°, with an acceleration voltage of 45 kV, tube current of 200 mA, and a scanning speed of 5°/min.

Fourier-transform infrared spectroscopy (FT-IR): The compositional analysis of the adhesive materials within the base layer of the sample Y2 samples was performed using a Fourier transform infrared spectrometer (Nicolet iS10, Thermo Scientific, Waltham, MA, USA), with a spectral resolution of 1 cm^−1^ across a wavenumber range of 400 to 4000 cm^−1^.

Pyrolysis–gas chromatography-mass spectrometry (Py-GC/MS): The system comprised a pyrolysis unit (EGA/PY-3030D, Frontier Labs, Koriyama, Japan) and a gas chromatograph mass spectrometer (GC/MS-QP2010 Ultra, Shimadzu, Kyoto, Japan). Approximately 0.2 mg of powdered samples were placed in a pyrolysis cup and reacted with 3 μL of 25% aqueous tetramethylammonium hydroxide (TMAH, Aladdin, Shanghai, China) for one hour. The samples were subjected to pyrolysis under infrared radiation at 600 °C, and the resultant chemical compounds were identified utilizing the NIST14 and other mass spectrometry databases.

## Figures and Tables

**Table 1:** Layer structure and compositions of cross-sections of the pigments.

Sample	Color Appearance	Analysis Methods	Elements
Y1	Red	SEM-EDS, m-RS, PLM Py-GC/MS, FT-IR, XRD	C, O, Al, Si, Hg, S, K, Ca
Y2	Blue	SEM-EDS, m-RS, PLM	C, O, Na, Al, Si, S, Ca
	White	SEM-EDS, m-RS, PLM	C, O, Pb
	Black	SEM-EDS, m-RS, PLM	C, O, Al, Si, S, Pb, Ca
Y3	White	SEM-EDS, m-RS, PLM	C, O, Mg, Al, Si, Pb
	Black	SEM-EDS, m-RS, PLM	C, O, Mg, Al, Si, S, Pb
Y4	Green	SEM-EDS, m-RS, PLM	C, O, Cu, As
	Black	SEM-EDS, m-RS, PLM	C, O

**Table 2:** Compounds identified by Py-GC/MS in the total ion chromatogram of sample Y1.

Peak Number	Retention Time (min)	Area (%)	Identified Compounds
1	1.833	4.04	Acethydrazide
2	2.093	3.12	2-methyl-1-pentanol
3	3.042	4.35	Valeraldehyde
4	4.176	3.58	Toluene
5	4.618	2.72	1-Octene
6	7.132	2.51	Cyclohexanone
7	7.339	1.7	Glyceryl methyl ether
8	9.971	1.13	2-Octanone
9	10.412	0.89	Cycloheptanone
10	10.55	0.91	6-Heptenoic acid methyl ester
11	12.512	0.64	Caprylic acid methyl ester
12	15.217	0.87	Glycinamide monohydrochloride
13	15.633	0.37	1-Tetradecene
14	15.702	0.38	n-Nonadecane
15	16.096	1.58	Methyl 9-oxononanoate
16	16.176	3.21	Dimethyl suberate
17	17.049	18.37	Dimethyl azelate
18	19.875	19.95	Methyl palmitate
19	21.039	7.75	Methyl oleate
20	21.183	17.11	Methyl stearate

**Table 3:** Samples of information on color paintings from the Cangjie Temple.

	Sample Number	Color	Sample Image	Optical Microscopic Image
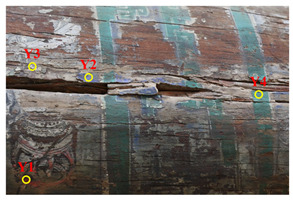	Y1	Red	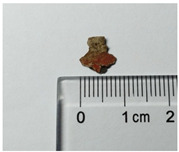	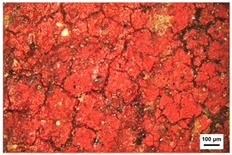
	Y2	Blue	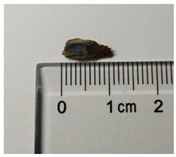	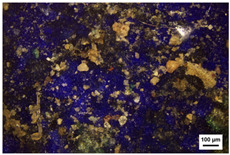
	Y3	White	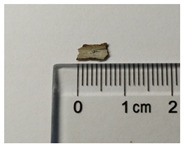	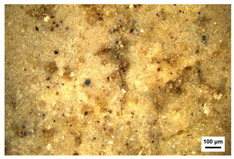
	Y4	Green	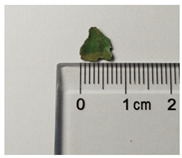	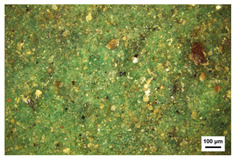

## Data Availability

The data presented in this study are available on request from the corresponding author.

## References

[1] Yang J., Tan F., Tan A. (2017). The Ancient Construction Materials and Methods: The Great Wall of China in Jinshanling as a Case Study. J. Constr. Eng. Proj. Manag..

[2] Pozo-Antonio J.S., Cardell C., Sánchez S., Rueda J. (2022). Reflectance of Oil Paintings: Influence of Paint Layer Thickness and Binder Amount. Coatings.

[3] Bersani D., Lottici P.P., Antonioli G., Campani E., Casoli A., Violante C. (2004). Pigments and binders in the wall paintings of Santa Maria della Steccata in Parma (Italy): The ultimate technique of Parmigianino. J. Raman Spectrosc..

[4] Siddall R. (2018). Mineral Pigments in Archaeology: Their Analysis and the Range of Available Materials. Minerals.

[5] Bersani D., Lottici P.P. (2016). Raman spectroscopy of minerals and mineral pigments in archaeometry: Raman spectroscopy of minerals in archaeometry. J. Raman Spectrosc..

[6] López-Montes A., Blanc R., Espejo T., Huertas-Perez J., Navalón A., Vílchez J. (2007). Simultaneous identification of natural dyes in the collection of drawings and maps from The Royal Chancellery Archives in Granada (Spain) by CE. Electrophoresis.

[7] Bin W., Hui Y., Bovyn G., Caen J. (2018). In situ investigation of Chinese export watercolours in the nineteenth century: Pigments and dyes. J. Inst. Conserv..

[8] Cheng C., Zhu Y., Zhang J., Li W., Teri G., Zheng L., Hu D. (2024). Mechanism for formation of porcine blood hydrogels used as additives in the mortar of traditional Chinese architectural painting. Herit. Sci..

[9] Elert K., Herrera A., Cardell C. (2017). Pigment-binder interactions in calcium-based tempera paints. Dyes Pigments.

[10] Bonaduce I., Ribechini E., Modugno F., Colombini M.P. (2016). Analytical Approaches Based on Gas Chromatography Mass Spectrometry (GC/MS) to Study Organic Materials in Artworks and Archaeological Objects. Top. Curr. Chem..

[11] Madariaga J.M. (2015). Analytical chemistry in the field of cultural heritage. Anal. Methods.

[12] Kopecká I., Svobodová E. (2014). Methodology for infrared spectroscopy analysis of sandwich multilayer samples of historical materials. Herit. Sci..

[13] Lobon M.G., Ghirardello M., Darder E.J., Cabello C.P., Bauza M., Cotte M., Burnstock A., Nevin A., Amato S.R., Izzo F.C. (2023). A study of cadmium yellow paints from Joan Miro’s paintings and studio materials preserved at the Fundacio Miro Mallorca. Herit. Sci..

[14] Zelinská J., Kopecká I., Svobodová E., Milovská S., Hurai V. (2018). Stratigraphic EM-EDS, XRF, Raman and FT-IR analysis of multilayer paintings from the Main Altar of the St. James Church in Levota (Slovakia). J. Cult. Herit..

[15] Franquelo M.L., Duran A., Castaing J., Arquillo D., Perez-Rodriguez J.L. (2012). XRF, μ-XRD and μ-spectroscopic techniques for revealing the composition and structure of paint layers on polychrome sculptures after multiple restorations. Talanta.

[16] Cheng X., Yang Q. (2015). Micro-Raman spectroscopy study of three green pigments containing Copper and Arsenic Copper and Arsenic. Sci. Conserv. Archaeol..

[17] Lau D., Villis C., Furman S., Livett M. (2008). Multispectral and hyperspectral image analysis of elemental and micro-Raman maps of cross-sections from a 16th century painting. Anal. Chim. Acta.

[18] Mugnaini S., Bagnoli A., Bensi P., Droghini F., Scala A., Guasparri G. (2006). Thirteenth century wall paintings under the Siena Cathedral (Italy). Mineralogical and petrographic study of materials, painting techniques and state of conservation. J. Cult. Herit..

[19] Wang X., Zhen G., Hao X., Tong T., Ni F., Wang Z., Jia J., Li L., Tong H. (2020). Spectroscopic investigation and comprehensive analysis of the polychrome clay sculpture of Hua Yan Temple of the Liao Dynasty. Spectrochim. Acta A.

[20] Spyros A., Anglos D. (2006). Studies of organic paint binders by NMR spectroscopy. Appl. Phys. A-Mater..

[21] Colombini M.P., Andreotti A., Bonaduce I., Modugno F., Ribechini E. (2010). Analytical Strategies for Characterizing Organic Paint Media Using Gas Chromatography/Mass Spectrometry. Acc. Chem. Res..

[22] Peifan Q., Deqi Y., Qi M., Aijun S., Jingqi S., Zengjun Z., Jianwei H. (2020). Study and Restoration of the Yi Ma Wu Hui Layer of the Ancient Coating on the Putuo Zongcheng Temple. Int. J. Archit. Herit..

[23] Matteini P., Camaiti M., Agati G., Baldo M.-A., Muto S., Matteini M. (2009). Discrimination of painting binders subjected to photo-ageing by using microspectrofluorometry coupled with deconvolution analysis. J. Cult. Herit..

[24] Ren F., Atlasevich N., Baade B., Loike J., Arslanoglu J. (2015). Influence of pigments and protein aging on protein identification in historically representative casein-based paints using enzyme-linked immunosorbent assay. Anal. Bioanal. Chem..

[25] Wang X., Zhen G., Hao X., Zhou P., Wang Z., Jia J., Gao Y., Dong S., Tong H. (2021). Micro-Raman, XRD and THM-Py-GC/MS analysis to characterize the materials used in the Eleven-Faced Guanyin of the Du Le Temple of the Liao Dynasty, China. Microchem. J..

[26] Liu J., Wei L.X., Wang Q., Lu Y.F., Zhang F., Shi J.Z., Li C., Cherian M.G. (2018). A review of cinnabar (HgS) and/or realgar (As_4_S_4_)-containing traditional medicines. J. Ethnopharmacol..

[27] Souto J., Gutiérrez-Vicente V., Prieto A.C. (2016). Raman analysis of Gothic wall paintings in the apse of the Santiago Apostol church in Alcazaren. J. Cult. Herit..

[28] Han K.Z., Yang H., Teri G., Hu S.S., Li J.X., Li Y.L., Ma E., Tian Y.X., Fu P., Luo Y.J. (2023). Spectroscopic Investigation of a Color Painting on an Ancient Wooden Architecture from the Taiping Heavenly Kingdom Prince Dai’s Mansion in Jiangsu, China. Minerals.

[29] Zhou G. (2010). Cinnabar in China and its developm ent in function. Dunhuang Res..

[30] Teri G., Han K., Huang D., Li Y., Tian Y., Chao X., Jia Z., Fu P., Li Y. (2023). A Study on the Materials Used in the Ancient Architectural Paintings from the Qing Dynasty Tibetan Buddhist Monastery of Puren, China. Materials.

[31] Dong S.H., Xiang J.K., Ji J., Wang Y.J., Zhang G., Fu P., Han J.W., Li L. (2023). Multi-Method Analysis of Painting Materials in Murals of the North Mosque (Linqing, China). Coatings.

[32] Sultan S., Kareem K., He L., Simon S. (2017). Identification of the authenticity of pigments in ancient polychromed artworks of China. Anal. Methods.

[33] Daniel F., Mounier A., Ricarrère P. Of some blue and bluish grey pigments in medieval mural paintings in the South West of France. Proceedings of the 39th International Symposium for Archaeometry.

[34] Brøns C., Hedegaard S.B., Bredal-Jørgensen J., Buti D., Pastorelli G. (2020). The rarest blue: An exceptional find of lapis lazuli in the polychromy of a funerary portrait from ancient Palmyra. Archaeometry.

[35] Zou W.H., Yeo S.Y., Cheng P., Zuo X.D., Zhao P., Li S.J. (2024). Unveiling the microstructure, materials, and painting period of ancient wall paintings at Shanxi Xianqing Temple, China. Archaeol. Anthrop. Sci..

[36] Wang J. (1996). Study on lapis lazuli pigment in Dunhuang, Maiji Mountain and Bingling Temple grottoes. Archaeology.

[37] Palamara E., Das P.P., Nicolopoulos S., Tormo Cifuentes L., Kouloumpi E., Terlixi A., Zacharias N. (2021). Towards building a Cathodoluminescence (CL) database for pigments: Characterization of white pigments. Herit. Sci..

[38] Frost R.L., Martens W., Kloprogge J.T., Ding Z. (2003). Raman spectroscopy of selected lead minerals of environmental significance. Spectrochim. Acta A.

[39] Bell I.M., Clark R.J.H., Gibbs P.J. (1997). Raman spectroscopic library of natural and synthetic pigments (pre-≈1850 AD). Spectrochim. Acta A.

[40] Fu P., Teri G., Li J., Huo Y., Yang H., Li Y. (2020). Analysis of an Ancient Architectural Painting from the Jiangxue Palace in the Imperial Museum, Beijing, China. Anal. Lett..

[41] Pfaff G. (2022). Carbon black pigments. Phys. Sci. Rev..

[42] Lampakis D., Karapanagiotis I., Katsibiri O. (2016). Spectroscopic Investigation Leading to the Documentation of Three Post-Byzantine Wall Paintings. Appl. Spectrosc..

[43] Cataldo F. (2000). A Raman study on radiation-damaged graphite by γ-rays. Carbon.

[44] Reich S., Thomsen C. (2004). Raman spectroscopy of graphite. Philos. T. R. Soc. A.

[45] Teri G., Fu P., Han K., Li J., Li Y., Jia Z., Wang Y., Li Y. (2022). Color Paintings of Taiping Heavenly Kingdom Royal Residence: An Analytical Study. Coatings.

[46] Zólyomi V., Koltai J., Kürti J. (2011). Resonance Raman spectroscopy of graphite and graphene. Phys. Status Solidi B.

[47] Coccato A., Jehlicka J., Moens L., Vandenabeele P. (2015). Raman spectroscopy for the investigation of carbon-based black pigments. J. Raman Spectrosc..

[48] Petrova O., Pankin D., Povolotckaia A., Borisov E., Krivul’ko T., Kurganov N., Kurochkin A. (2019). Pigment palette study of the XIX century plafond painting by Raman spectroscopy. J. Cult. Herit..

[49] D’Imporzano P., Batur K., Keune K., Koornneef J.M., Hermens E., Noble P., Van Zuilen K., Davies G.R. (2021). Lead isotope heterogeneity in lead white: From lead white raw pigment to canvas. Microchem. J..

[50] Burgio L., Clark R.J.H., Firth S. (2001). Raman spectroscopy as a means for the identification of plattnerite (PbO_2_), of lead pigments and of their degradation products. Analyst.

[51] Svarcová S., Hradil D., Hradilová J., Cermáková Z. (2021). Pigments-copper-based greens and blues. Archaeol. Anthrop. Sci..

[52] Keune K., Boon J.J., Boitelle R., Shimadzu Y. (2013). Degradation of Emerald green in oil paint and its contribution to the rapid change in colour of the Descente des vaches(1834–1835) painted by Théodore Rousseau. Stud. Conserv..

[53] Hawa A., Salaemae P., Prachasaree W., Tonnayopas D. (2017). Compressive Strength and Microstructural Characteristics of Fly Ash based Geopolymer with High Volume Field Para Rubber Latex. Rev. Rom. Mater..

[54] Barone G., Fugazzotto M., Mazzoleni P., Raneri S., Russo A. (2019). Color and painting techniques in Etruscan architectural slabs. Dyes Pigments.

[55] Gelzo M.G.M., Vergara A. (2014). Comparison of binder compositions in Pompeian wall painting styles from *Insula Occidentalis*. Chem. Cent. J..

[56] Salvadó N., Buti S., Pantos E., Bahrami F., Labrador A., Pradell T. (2008). The use of combined synchrotron radiation micro FT-IR and XRD for the characterization of Romanesque wall paintings. Appl. Phys. A-Mater..

[57] Dallongeville S., Garnier N., Rolando C., Tokarski C. (2015). Proteins in Art, Archaeology, and Paleontology: From Detection to Identification. Chem. Rev..

[58] Zhou Z.B., Shen L., Li C.L., Wang N., Chen X.L., Yang J., Zhang H. (2020). Investigation of gilding materials and techniques in wall paintings of Kizil Grottoes. Microchem. J..

[59] Barberis E., Manfredi M., Marengo E., Zilberstein G., Zilberstein S., Kossolapov A., Righetti P.G. (2019). Leonardo’s Donna Nuda unveiled. J. Proteomics.

[60] Sefcu R., Pitthard V., Chlumská S., Turková I. (2017). A multianalytical study of oil binding media and pigments on Bohemian Panel Paintings from the first half of the 14th century. J. Cult. Herit..

[61] Wang N., He L., Zhao X., Simon S. (2015). Comparative analysis of eastern and western drying-oil binding media used in polychromic artworks by pyrolysis–gas chromatography/mass spectrometry under the influence of pigments. Microchem. J..

